# Antibody-drug conjugates targeting HER2 for the treatment of urothelial carcinoma: potential therapies for HER2-positive urothelial carcinoma

**DOI:** 10.3389/fphar.2024.1326296

**Published:** 2024-03-20

**Authors:** Chia-Hsien Shih, Yu-Hua Lin, Hao-Lun Luo, Wen-Wei Sung

**Affiliations:** ^1^ School of Medicine, Chung Shan Medical University, Taichung, Taiwan; ^2^ Division of Urology, Department of Surgery, Cardinal Tien Hospital, New Taipei City, Taiwan; ^3^ Department of Chemistry, Fu Jen Catholic University, New Taipei City, Taiwan; ^4^ Graduate Institute of Biomedical and Pharmaceutical Science, Fu Jen Catholic University, New Taipei City, Taiwan; ^5^ Department of Urology, Kaohsiung Chang Gung Memorial Hospital and Chang Gung University College of Medicine, Kaohsiung, Taiwan; ^6^ Center for Shockwave Medicine and Tissue Engineering, Kaohsiung Chang Gung Memorial Hospital and Chang Gung University College of Medicine, Kaohsiung, Taiwan; ^7^ Department of Urology, Chung Shan Medical University Hospital, Taichung, Taiwan; ^8^ Institute of Medicine, Chung Shan Medical University, Taichung, Taiwan

**Keywords:** bladder neoplasm, metastasis, human epidermal growth factor receptor, ErbB2, systemic therapy

## Abstract

Urothelial carcinoma (UC) is a common cancer characterized by high morbidity and mortality rates. Despite advancements in treatment, challenges such as recurrence and low response rates persist. Antibody-drug conjugates (ADCs) have emerged as a promising therapeutic approach for various cancers, although their application in UC is currently limited. This review focuses on recent research regarding ADCs designed to treat UC by targeting human epidermal growth factor receptor 2 (HER2), a surface antigen expressed on tumor cells. ADCs comprise three main components: an antibody, a linker, and a cytotoxic payload. The antibody selectively binds to tumor cell surface antigens, facilitating targeted delivery of the cytotoxic drug, while linkers play a crucial role in ensuring stability and controlled release of the payload. Cleavable linkers release the drug within tumor cells, while non-cleavable linkers ensure stability during circulation. The cytotoxic payload exerts its antitumor effect by disrupting cellular pathways. HER2 is commonly overexpressed in UCs, making it a potential therapeutic target. Several ADCs targeting HER2 have been approved for cancer treatment, but their use in UC is still being tested. Numerous HER2 ADCs have demonstrated significant growth inhibition and induction of apoptosis in translational models of HER2-overexpressing bladder cancer. Ongoing clinical trials are assessing the efficacy and safety of ADCs targeting HER2 in UC, with the aim of determining tumor response and the potential of ADCs as a treatment option for UC patients. The development of effective therapies with improved response rates and long-term effectiveness is crucial for advanced and metastatic UC. ADCs targeting HER2 show promise in this regard and merit further investigation for UC treatment.

## Introduction

Urothelial carcinoma (UC) is the sixth most common cancer in adults, with 573,258 new cases and 212,536 deaths reported worldwide in 2020 (Sung et al., 2021). UC originates from lesions in the urothelial epithelium, commonly affecting the bladder and urethra. Non-muscle invasive bladder carcinoma (NMIBC) accounts for approximately 75% of UC diagnoses, while muscle invasive bladder carcinoma (MIBC) accounts for 20% (Alfred Witjes et al., 2017; Luo et al., 2022). The five-year survival rate is over 90% for NMIBC but drops to 50% for MIBC (Alfred Witjes et al., 2017).

The primary treatment for NMIBC involves transurethral resection of the bladder tumor, often followed by chemotherapy or infusion therapy with *bacillus* Calmette-Guérin (BCG). However, NMIBC still presents a significant recurrence rate, with reported rates ranging from 50% to 70% within 5 years. MIBC patients are typically treated with radical cystectomy and bilateral pelvic lymphadenectomy, with cisplatin-based neoadjuvant chemotherapy (NAC) recommended due to high relapse rates following cystectomy (Apolo et al., 2014). The use of NAC significantly reduces mortality rates and improves five-year survival. Chemotherapy and immunotherapy are suggested for patients who are ineligible for cisplatin. Recent studies have shown that neoadjuvant atezolizumab (ABACUS), pembrolizumab (PURE-01), and nivolumab (nivo) plus ipilimumab can achieve complete pathologic response rates of approximately 30% to 40% (Zucali et al., 2020). Despite advancements in UC treatment, response rates remain low, and the majority of patients experience eventual relapse. Therefore, there is a need to develop therapies that are more effective and provide long-term response for advanced and metastatic urothelial carcinoma (mUC) (Yin et al., 2016; Osanto and Álvarez Gómez de Segura, 2020; Roviello et al., 2022).

With the establishment of the Cancer Genome Atlas (TCGA) for UC, there has been rapid development in the field of molecularly targeted therapy, specifically in identifying mutant tumor antigens. Among these advancements, antibody-drug conjugates (ADCs) have emerged as a promising cancer therapy. ADCs involve the combination of cytotoxic agents with antibodies that specifically target surface antigens on tumor cells, allowing for selective delivery of the cytotoxic agents into the tumor cells. Currently, ADCs are primarily utilized in the treatment of breast cancer and malignant lymphoma. The first ADC to gain approval from the US Food and Drug Administration (FDA) was gemtuzumab ozogamicin, which is used for the treatment of acute myeloid leukemia. Subsequently, trastuzumab emtansine (T-DM1) was approved as a clinical drug for the treatment of human epidermal growth factor receptor 2 (HER2)-positive breast cancer. While the development of ADCs has progressed significantly, their application in UC remains limited (Sievers and Senter, 2013; Diamantis and Banerji, 2016; Drake and Rabuka, 2017). However, the FDA has recently approved enfortumab vedotin in 2019 and sacituzumab govitecan in 2021 for the treatment of UC, showcasing the promising potential of ADC research in the field of urology (Hanna et al., 2022). This review focuses on recent research regarding ADCs targeting HER2, an antigen expressed on UC tumor cells, for the treatment of UC.

## ADCs

**FIGURE 1:**
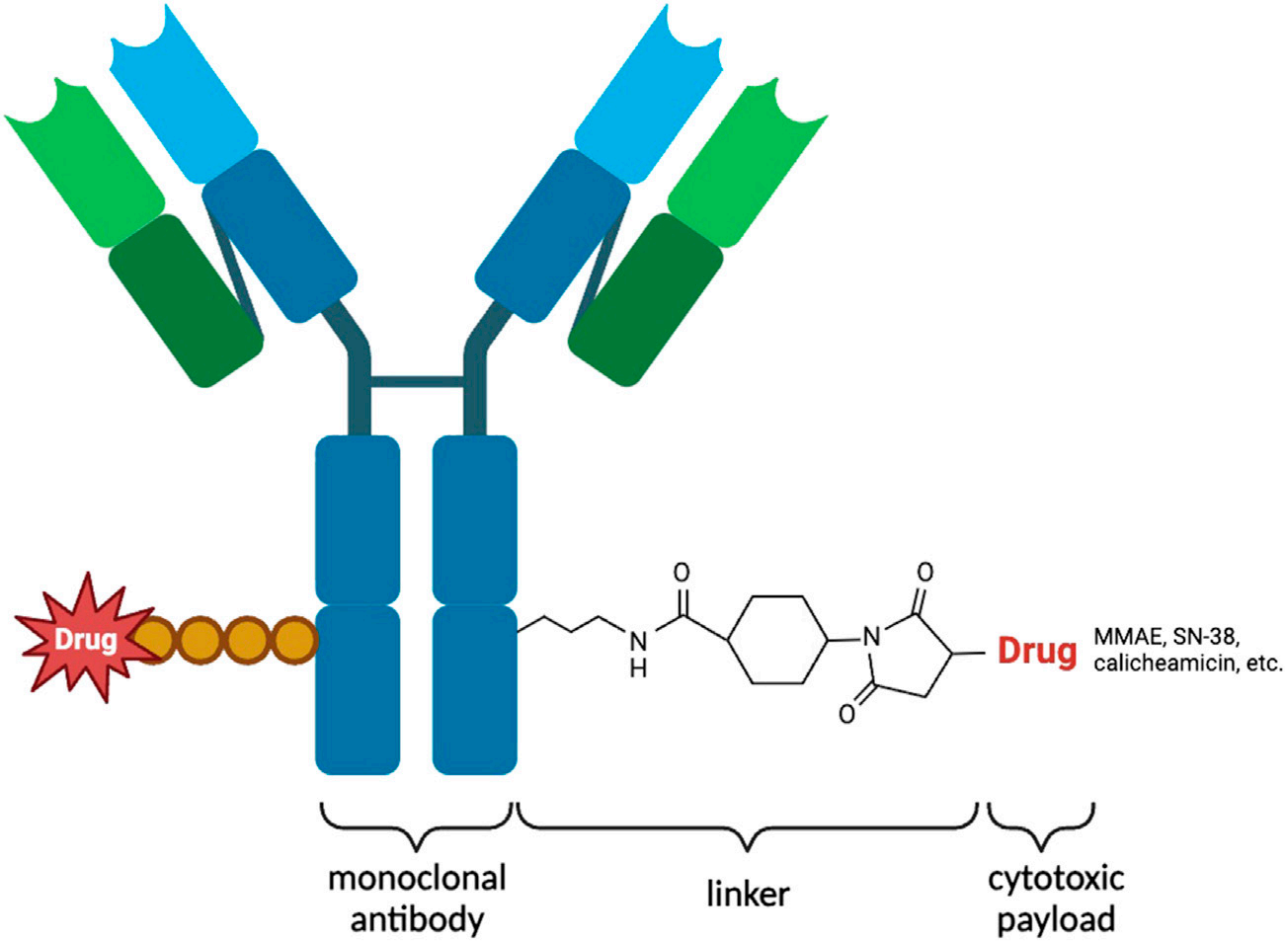
Illustration of the structure of an ADC used for targeted therapy. The ADC consists of heavy and light chains that form an antibody capable of specifically recognizing antigens on the surface of tumor cells. Cytotoxic payloads are attached to the antibody through linkers. ADCs are internalized into tumor cells through receptor-mediated endocytosis, where proteases cleave the linkers, releasing the cytotoxic payloads. These payloads induce cell death specifically within the targeted tumor cells. ADCs combine the specificity of antibodies with potent cytotoxic agents, providing a promising approach for targeted cancer therapy. By delivering cytotoxic payloads directly to tumor cells, ADCs enhance therapeutic efficacy while minimizing systemic toxicity.

**FIGURE 2:**
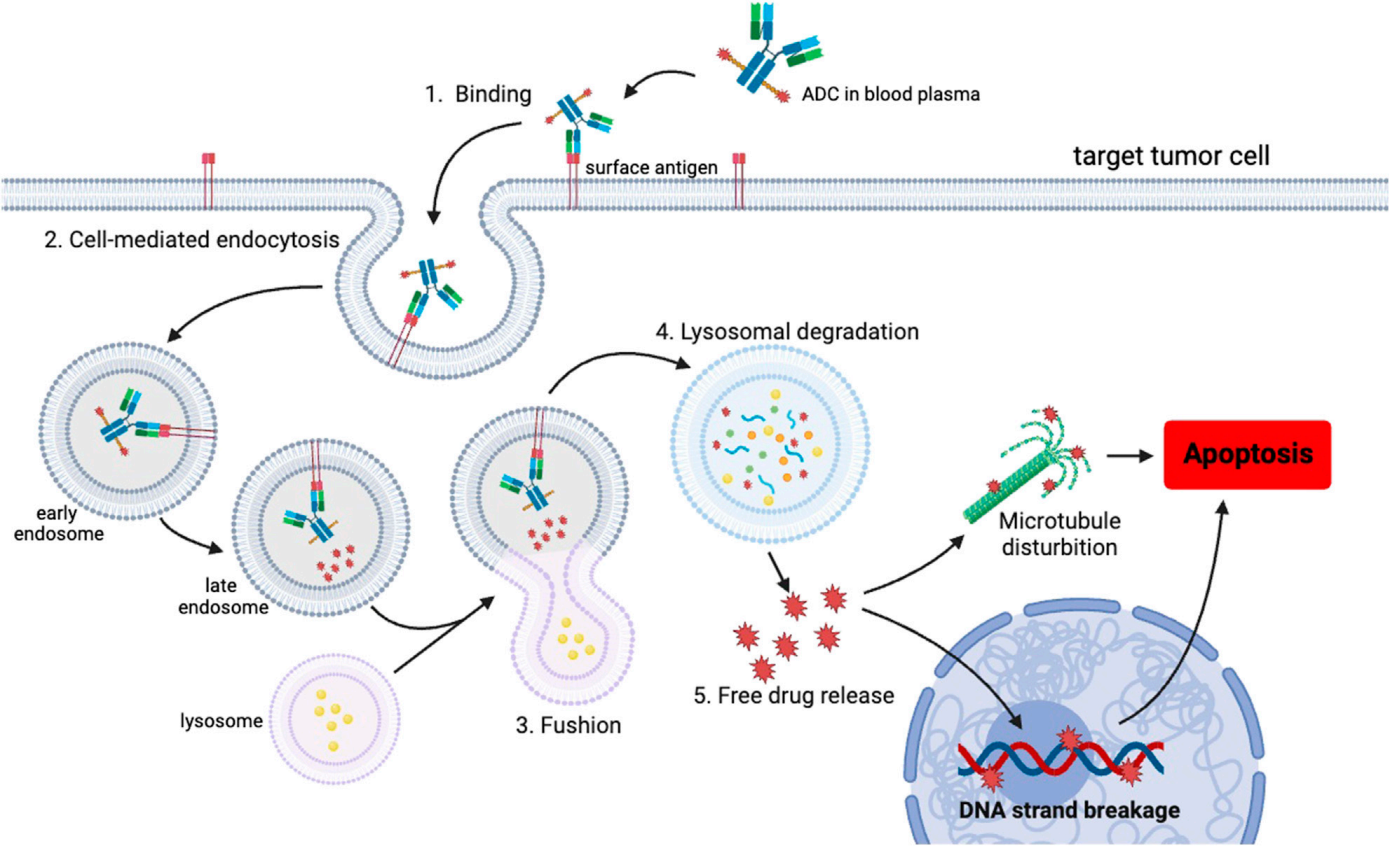
Schematic representation of the uptake of the ADC and the subsequent release of the cytotoxic payload within a cancer cell. The mechanism of action involves several steps, initiating with the binding of the ADC to the target antigen on the cell surface, followed by internalization through endocytosis. Once internalized, the ADCs are initially transported to early endosomes and subsequently to late endosomes. Subsequently, late endosomes trigger the fusion of vesicles containing ADCs with lysosomes. Within the lysosomes, the ADCs undergo degradation, leading to the release of cytotoxic drugs inside the cells. Ultimately, the liberated drugs induce apoptosis specifically in the targeted tumor cells. This targeted mechanism enables precise and efficient delivery of therapeutic payloads.

An ADC is an innovative therapeutic approach that consists of three key components (Figure 1). ADCs primarily consist of antibodies, linkers, and payloads. By combining cytotoxic drugs with highly specific antibodies that target surface antigens of specific tumor cells, ADCs are able to selectively kill cancer cells while significantly reducing side effects (Marei et al., 2022). Most ADCs are administered intravenously and follow a similar mechanism for releasing cytotoxic drugs in the body. They reach the target tissue through the cardiovascular system and enter tumor cells via receptor-mediated endocytosis. Once inside the tumor cells, ADCs form early endosomes, which later mature into late endosomes and merge with lysosomes. ADCs with cleavable linkers detach from early or secondary endosomes and subsequently release potent cytotoxic drugs. On the other hand, ADCs with non-cleavable linkers need to fuse with lysosomes before releasing the cytotoxic drugs. Ultimately, the disruption of vital cellular biosynthetic pathways leads to the destruction of cancer cells (Walsh et al., 2021) (Figure 2).

## Antibody

A critical step in ADC design is the selection of stable and highly antigen-specific antibodies to effectively deliver ADCs to the intended site. These antibodies must be capable of distinguishing the surface antigens of tumor cells from normal cells. Otherwise, there is a risk of delivering ADCs to healthy tissue, leading to off-target toxicity, or the antibodies may undergo degradation by the immune system before reaching the tumor cells. Currently, there are licensed ADCs targeting potent antigens such as HER2, CD33/30/22, Trop-2, and Nectin4 for the treatment of malignancies (Lattanzi and Rosenberg, 2020). In the development of ADCs, SLITRK6 is also utilized as a target due to its expression in multiple epithelial tumors, including bladder, lung, and breast cancer. The most potent ADC known as ASG-15ME is currently undergoing phase I clinical trials for the treatment of mUC (Morrison et al., 2016). Among the various types of antibodies used, humanized IgG, particularly IgG1, is the most commonly employed. The use of antibodies derived from mice poses potential risks, which is why the adoption of humanized antibodies has significantly minimized these risks (Nagayama et al., 2017).

## Linker

Linkers play a crucial role in the pharmacokinetics, drug efficacy, and safety of ADCs. It is imperative for the stability of linkers within the circulatory system to prevent the premature release of cytotoxic drugs from ADCs, as this may result in off-target toxicity. However, these linkers must be designed to be unstable under specific conditions to facilitate the delivery of the drug payload into tumor cells. There are two main types of linkers: cleavable and non-cleavable. Cleavable linkers undergo hydrolysis, protease cleavage, or disulfide bond cleavage within early endosomes or secondary endosomes, allowing the release of potent cytotoxic drugs. A commonly utilized mechanism involves the action of glutathione, where cleavable linkers are designed to be sensitive to glutathione. This sensitivity leads to linker cleavage, subsequently releasing the therapeutic payload into the intracellular environment. This mechanism capitalizes on the reducing properties of glutathione, facilitating controlled drug release specifically within the intracellular space (Baah et al., 2021). Examples of ADCs utilizing this cleavable linker mechanism include brentuximab vedotin, enfortumab vedotin, and polatuzumab vedotin, which employ a pH-sensitive hydrazone linker (Sonawane et al., 2017). On the other hand, non-cleavable linkers are resistant to cleavage and remain intact after being fused to the lysosome. The acidic environment generated by the lysosomal proton pump promotes protein degradation through cathepsin B or plasmin-mediated pathways. Studies have demonstrated that non-cleavable linkers exhibit greater stability in blood compared to cleavable linkers (Jain et al., 2015). Furthermore, ADCs with non-cleavable linkers present a reduced risk of systemic toxicity caused by premature payload release when the drug payload is released within the lysosome. The FDA has approved T-DM1 and mafodotin belantamab as examples of ADCs utilizing non-cleavable linkers (Tong et al., 2021).

## Payload

The payload in this context refers to a cytotoxic drug that is released in high concentrations within the tumor and possesses antitumor effects. Generally, payloads can be categorized into two groups based on their mechanism of cytotoxicity: microtubule disruptors and DNA damage agents. Auristatin, specifically monomethyl auristatin E (MMAE), is a synthetic antitumor drug used as a cytotoxic payload in ADCs. MMAE, an antimitotic drug, induces G2/M cell cycle arrest and apoptosis by inhibiting tubulin assembly. Maytansinoids, which are macrolides belonging to the ansamycin group, can be extracted from plants of the Maytenus genus. They share a similar function with MMAE (Sapra and Shor, 2013; Bouchard et al., 2014). DNA-damaging agents can exert their effects during various phases of the cell cycle. Calichemycin, for instance, is a DNA-damaging agent that binds to the minor groove of DNA sequences, thereby inhibiting DNA replication. Sacituzumab govitecan delivers camptothecin analogues, like SN-38, as a payload. SN-38 inhibits topoisomerase I, leading to DNA damage and breakage (Goldenberg and Sharkey, 2019). The payload of gemtuzumab ozogamicin is calicheamicin. Dokamycin, similar to calicheamicin, alters the DNA structure by binding to the minor groove of DNA and causing DNA alkylation (Sapra et al., 2011; Kern et al., 2016).

## Surface antigen of tumor cells: HER2

HER2 is a receptor tyrosine kinase expressed on the cell membrane surface that plays a role in signaling pathways governed by the proto-oncogene HER2/neu. These pathways are responsible for cell growth and differentiation. The HER2 gene is located on the long arm of human chromosome 17 (17q21-q22) and functions as a proto-oncogene (Schechter et al., 1984). The downstream signaling of HER2 occurs when HER family members dimerize with other ligands, leading to phosphorylation and activation of the PI3K/AKT and MEK/ERK pathways. Mutations in the HER2 pathway can trigger oncogenesis by enhancing dimerization and autophosphorylation, resulting in uncontrolled cell growth. Overexpression of HER2 due to gene amplification leads to cell proliferation, inhibition of apoptosis, and angiogenesis, which are major factors contributing to aggressive carcinoma (Hansel et al., 2008; Fleischmann et al., 2011).

The highest prevalence of HER2 overexpression is observed in breast cancer tissue. HER2-positive breast cancer comprises approximately 15% to 20% of all breast carcinoma cases and is characterized by its highly aggressive nature and poor prognosis. These cancer cells exhibit not only strong proliferative abilities but also resistance to certain chemotherapy drugs. Even after undergoing surgical treatment, there remains a high likelihood of cancer cell recurrence and metastasis, resulting in limited long-term survival for patients (Gámez-Chiachio et al., 2022). Therefore, HER2 plays a pivotal role in cancer screening and targeted treatment. Numerous HER2-targeted therapies have been specifically developed, including tyrosine kinase inhibitors (TKIs) and monoclonal antibodies (mAbs). TKIs such as lapatinib, neratinib, and tucatinib can bind to the intracellular tyrosine kinase domain of HER2, effectively blocking the HER2-mediated intracellular signaling pathways. Trastuzumab, a type of mAb, functions by inhibiting receptor dimerization through binding to the extracellular region of HER2 (Perez et al., 2014). The addition of trastuzumab has shown a 37% improvement in overall patient survival and a 40% improvement in disease-free survival (Chumsri et al., 2019).

HER2 is also overexpressed in UC, and targeting HER2 in UC has gained popularity in recent years in clinical research. Therapies aimed at HER2 have become a standard practice for treating HER2-positive breast carcinoma, and the potential for HER2 as a therapeutic target in UC is currently being evaluated. Several ongoing clinical phase II and III trials are assessing the effectiveness of HER2-targeted therapies for UC patients. According to a study conducted by M. Laé, 5.1% of invasive bladder carcinomas exhibited HER2 gene amplification, while HER2 overexpression was observed in 9.2% of tumor samples (Laé et al., 2010). Another study by J. Tschui revealed that approximately three-quarters of aggressive HER2 gene-amplified subtypes of bladder cancer showed HER2 protein overexpression (Tschui et al., 2015). These findings suggest that HER2 overexpression in bladder carcinomas is a result of HER2 gene amplification, highlighting HER2 as a promising candidate for targeted therapy.

Unlike traditional Herceptin treatment, which specifically targets HER2-amplified tumor cells, ADC therapy appears to exert its effects independently of gene amplification. Remarkably, a substantial proportion of HER2 protein expression is detected even in the absence of gene amplification, which is also observed in malignant urothelial cells (Scherrer et al., 2022). The important feature that sets ADC therapy apart from traditional therapy is its unique ability to diffuse its effects to HER2-expressing tumor cells regardless of their gene amplification status. This diffusion effect represents a critical advantage, as it expands the therapeutic range to encompass tumor cells with lower levels or no HER2 expression (Lei et al., 2023). Consequently, ADC presents a promising approach for addressing the challenge of intratumoral heterogeneity, which has been a significant hurdle in achieving favorable treatment outcomes for invasive bladder cancer. The limitations of traditional Herceptin treatment for invasive bladder cancer highlight the importance of seeking alternative strategies that can effectively target tumors with heterogeneous HER2 expression (Jiang et al., 2020).

The diffusion effect of ADC therapy allows it to penetrate and eliminate HER2-expressing tumor cells throughout the tumor, minimizing the chances of treatment resistance and disease recurrence associated with incomplete targeting.

## Challenges of manufacturing ADCs

The production of ADCs presents multifaceted challenges in pharmaceutical manufacturing sine ADCs are heterogeneous mixture of compounds with varying number of drugs and multiple conjugation sites. Therefore, a Design of Experiments (DoE) approach is typically used to determine the impact of single variables as well as interactions of multiple variables in early development stages. Initial stages involve the optimization of monoclonal antibodies (mAbs) for robustness in the ADC manufacturing environment, with a focus on physicochemical properties ensuring suitability for the process (Ducry, 2012).

AS for the ADC process development, achieving the desired drug-antibody-ratio (DAR) is paramount for efficacy and regulatory compliance. It is necessary to take consideration of potential challenges such as mAb aggregation and drug/linker adverse effects. The manufacturing environment demands adherence to Current Good Manufacturing Process (cGMP) standards, emphasizing aseptic conditions to protect operators and products. The challenges escalate when handling powdered cytotoxic reagents, requiring specialized containment measures and advanced personal protection equipment. Current manufacturing plants is designed for reducing microbial contamination such as endotoxin, but not able to eliminate highly potent cytotoxic compound with occupational exposure levels (OELs) in the nanogram range (Magalhães et al., 2007). Moreover, the purification process introduces additional complexities, including variations in DAR, unincorporated drug, spacer derivatives, and organic solvents. Tangential flow filtration (TFF) and chromatography modalities are employed, necessitating optimization to account for potential stability and solubility issues. Throughout, operator and environmental protection remain paramount, leading to the adoption of closed systems and single-use components which cause production cost increasing.

In summary, ADC manufacturing presents complex challenges requiring innovations in mAb processing, conjugation chemistry, purification techniques, and facility design. Success in overcoming these challenges is essential for the development and production of safe, effective, and marketable ADC therapies.

## Anti-HER2 monoclonal antibodies

The activation of HER2 involves the formation of dimeric or oligomeric structures, and one of the mechanisms is thought to be influenced by HER2 overexpression. HER2 are found in exceptionally high levels on the tumor cell surface, often thousands of times more than in normal cells, and HER2 is constitutively activated or phosphorylated (Albanell et al., 2003). Studies conducted with wild-type neu (rat homologue of human HER2) have revealed that when present in high concentrations, neu tend to exist as dimers or even larger multimeric complexes. This observation suggests that when human HER2 is overexpressed, the receptors can similarly form dimeric or oligomeric HER2 structures (homodimerization) (Samanta et al., 1994). Since HER2 is a ligand orphan receptor, this process doesn't rely on the presence of a specific ligand to trigger. Activation process can occur spontaneously due to the elevated concentration of HER2 at the cell surface.

Trastuzumab is a monoclonal humanized anti-HER2 mAb which binds to domain IV of the extracellular segment and blocks the cleavage of the extracellular domain of HER2. The binding of Trastuzumab will subsequently induce its antibody-induced receptor downregulation or inhibit HER2-mediated intracellular signaling cascades. These mechanisms cause disruption of receptor dimerization and phosphorylation, which made tumor cells arrest in the G1 phase (Gemmete and Mukherji, 2011). Otherwise, Trastuzumab also conduct the activation of antibody-dependent cell-mediated cytotoxicity (ADCC) by attracting immune cells such as natural killer (NK) cells or dendritic cells (DCs). Immune cells can effectively approach tumor sites and induce immune responses by binding to the Fc domain of Trastuzumab (Swain et al., 2023). Trastuzumab display a potent antiproliferative effect against tumor cells with high expression of HER2, but their cytotoxic effects are limited. Therefore, the combination of anti-HER2 mAb with chemotherapy agents can produce higher anti-tumor effect. Additionally, HER2 overexpressed cells have a high proliferation rate, causing a significant response to cytotoxic drug treatment. Cytotoxic drugs exert strong destructive effects on tumor cells but also significantly impact every dividing cell, leading to severe adverse reactions. Cytotoxic adverse events caused by chemotherapies highlights the ability of ADCs to accurately deliver cytotoxic drugs into tumor cells.

## ADCs targeting HER2 in UC

HER2-amplified UC exhibits distinctive morphological characteristics, which makes HER2 a potential target for therapeutic interventions in UC (Patelli et al., 2022). The approval of several FDA-approved antibody-drug conjugates (ADCs) targeting HER2 for cancer treatment marks a significant milestone, and novel ADCs focusing on HER2 represent the most promising strategy in UC. However, it should be noted that nearly all ADCs intended for UC treatment are currently undergoing testing. In the following sections, we will provide a review of the novel ADCs targeting HER2 in UC that are currently being evaluated in clinical trials (Table 1).

**TABLE 1:** Ongoing clinical trials with HER-2 targeting ADCs.

ADC	Trial	Phase	Number of patients	Drugs involved	Condition or disease	Status
Trastuzumab emtansine (T-DM1)	NCT02999672	phase II	20	Trastuzumab Emtansine	Bladder Cancer, Pancreas Cancer, Cholangiocellular Carcinoma	Completed
	NCT02675829	phase II	140	Ado-Trastuzumab Emtansine	Solid Tumor Cancers, Lung Cancer, Bladder Cancer, Urinary Tract Cancers	Recruiting
	NCT04632992	phase II	252	Entrectinib, Inavolisib, Alectinib, Ipatasertib, Atezolizumab, Trastuzumab, Emtansine, Pertuzumab + Trastuzumab + Hyaluronidase-zzxf, Tucatinib, Investigator’s Choice of Chemotherapy, Paclitaxel, Tiragolumab, Pralsetinib	Advanced Unresectable or Metastatic Solid Malignancy	Active, not recruiting
Trastuzumab deruxtecan (T-DXd)	NCT04644068	phase II	604	AZD5305, Paclitaxel, Carboplatin, T-Dxd, Dato-DXd, Camizestrant	Ovarian Cancer, Breast Cancer, Pancreatic Cancer, Prostate Cancer, Non-small Cell Lung Cancer, Small Cell, Lung Cancer, Colorectal Cancer, Bladder Cancer, Gastric Cancer, Biliary Cancer, Cervical Cancer, Endometrial Cancer	Recruiting
	NCT04482309	phase II	468	Trastuzumab deruxtecan	Bladder Cancer, Biliary Tract Cancer, Cervical Cancer, Endometrial Cancer, Ovarian Cancer, Pancreatic Cancer, Rare Tumors	Active, not recruiting
Disitamab vedotin (RC48)	NCT04879329	phase II	332	disitamab vedotin, pembrolizumab	Urothelial Carcinoma	Recruiting
	NCT05495724	phase II	176	Disitamab Vedotin + Tislelizumab	Her2 Overexpressing High-Risk Non-Muscle Invasive Bladder Urothelial Carcinoma	Recruiting
	NCT05837806	phase II	27	Tislelizumab + Disitamab vedotin	Upper Tract Urothelial Carcinoma	Recruiting
	NCT03809013	phase II	60	Disitamab vedotin	HER2 Overexpressing Locally Advanced or Metastatic Urothelial Cancer	Active, not recruiting
	NCT04073602	phase II	19	Disitamab vedotin	Locally Advanced or Metastatic HER2-negative Urothelial Cancer	Active, not recruiting
	NCT05488353	phase IV	48	Disitamab Vedotin + Penpulimab	Cisplatin Intolerant CT2-T4anxm0 Bladder Urothelial Carcinoma	Not yet recruiting

## Trastuzumab emtansine (T-DM1, Kadcyla^®^)

T-DM1, the first approved antibody-drug conjugate (ADC) for the treatment of solid tumors, is administered following surgical treatment for HER2-positive early breast cancer (Lambert and Chari, 2014). Prior to its approval, the use of ADCs for solid tumor treatment encountered significant challenges. The tumor microenvironment presented biological barriers, such as dense stroma and inadequate blood vascularization, leading to reduced drug delivery and off-target toxicity (Criscitiello et al., 2021). T-DM1 utilizes trastuzumab, a humanized mAb that targets the HER2 protein on tumor cells. The trastuzumab and cytotoxic payload, DM1, are linked by a non-cleavable thioether linker, ensuring stability but relying on lysosomal degradation for payload release. DM1 binds to tubulin, disrupting the formation of microtubule networks, resulting in antimitotic toxicity and apoptotic cell death (Barok et al., 2014).

A study conducted by Tetsutaro Hayashi investigated the impact of T-DM1 compared to trastuzumab in various *in vitro* and *in vivo* models of HER2-overexpressing bladder cancer. The results demonstrated that T-DM1 exhibited significant growth inhibition when compared to trastuzumab. Additionally, T-DM1 induced apoptosis following G2/M cell cycle arrest specifically in the RT4V6 cell line, which showed the highest level of HER2 expression among all bladder cancer cell lines studied. Furthermore, the study revealed that cell lines with acquired cisplatin resistance exhibited higher HER2 expression, and these resistant cells demonstrated increased sensitivity to T-DM1 due to the induction of apoptosis. These findings suggest that T-DM1 holds great potential for antitumor effects in preclinical models of HER2-overexpressing BC (Hayashi et al., 2015).

There are two phase II trials currently evaluating the tumor response of T-DM1 in patients with HER2-overexpressing solid bladder cancer, and one of these trials has reported its results. The study with the identifier NCT02999672 enrolled 13 participants who had HER2-overexpressing advanced or metastatic urinary bladder cancer, pancreatic cancer, or cholangiocarcinoma. The main objectives of this study were to assess the efficacy, safety, and pharmacokinetics of T-DM1. The participants received T-DM1 through intravenous (IV) infusion, either as Regimen A (2.4 mg/kg, weekly) or Regimen B (3.6 mg/kg, every 3 weeks). The investigators evaluated the best overall response (BOR) using response evaluation criteria in solid tumors, where BOR was defined as achieving the best objective response, either complete response (CR) or partial response (PR). In this study, 38.5% of the participants with bladder cancer exhibited a reduction of more than 30% (defined as PR) in the sum of the diameters of the target tumors compared to the baseline sum diameter.

The NCT02675829 study, which is currently enrolling participants, is a clinical trial investigating the use of T-DM1 in patients with HER2-amplified or mutant cancers, including solid tumor cancers, lung cancer, bladder cancer, and urinary tract cancers. T-DM1 is administered intravenously at a dosage of 3.6 mg/kg every 21 days until disease progression or unacceptable toxicity occurs.

The NCT04632992 study aims to assess targeted therapies in individuals with advanced solid tumors exhibiting specific genomic alterations or protein expression patterns predictive of treatment response. This ongoing phase II trial examines the combination of TDM-1 with other novel drugs, such as tucatinib and atezolizumab.

## Trastuzumab deruxtecan (T-DXd, Enhertu^®^)

Trastuzumab deruxtecan (T-DXd) and T-DM1 are examples of trastuzumab-based drug conjugates, where trastuzumab is utilized as a mAb to construct an ADC. T-DXd consists of a cleavable tetrapeptide-based linker and a cytotoxic topoisomerase I inhibitor. It has demonstrated long-lasting antitumor activity in patients with HER2-positive metastatic breast cancer who have undergone two or more prior anti-HER2 treatments (Modi et al., 2020). Moreover, treatment with T-DXd has resulted in significant enhancements in response rate and overall survival (OS) among patients with HER2-positive gastric cancer (Shitara et al., 2020).

The study, NCT03523572, investigated the impact of T-DXd in combination with Nivolumab on patients with HER2-overexpressing UC. The phase Ib trial enrolled 34 patients and observed an objective response rate (ORR) of 36.7%, with a median duration of response of 13.1 months, median progression-free survival of 6.9 months, median time to response of 1.9 months, and median OS of 11.0 months. These primary findings suggest that the combination of T-DXd and nivo demonstrates antitumor activity in patients with HER2-overexpressing UC.

Although T-DXd has been approved for the treatment of metastatic HER2-positive breast cancer, as well as metastatic HER2-positive gastric and gastroesophageal junction adenocarcinoma, its effect on HER2 overexpressing UC is still being evaluated (Tong et al., 2021). The NCT04644068 phase II trial is currently investigating the safety, tolerability, and anticancer activity of AZD5305, a PARP inhibitor (Poly ADP-ribose Polymerase inhibitor), in patients with advanced solid cancers, including breast cancer, pancreatic cancer, prostate cancer, and bladder cancer. The study comprises individual modules, in which AZD5305 is administered as monotherapy or in combination with specific partners such as paclitaxel, carboplatin, T-DXd, or Dato-DXd.

The phase II trial NCT04482309 enrolled 268 participants diagnosed with various types of tumors, including BC, biliary cancer, cervical cancer, endometrial cancer, ovarian cancer, pancreatic cancer, and rare tumors. The objective of this study is to assess the effectiveness and safety of T-DXd in treating specific tumors that overexpress HER2.

## Disitamab vedotin (RC48)

Disitamab vedotin (RC48) is a novel antibody-drug conjugate (ADC) composed of a humanized mAb (hertuzumab), cytotoxic MMAE, and a mc-val-cit-PABC linker (Yu et al., 2022). Compared to trastuzumab, hertuzumab exhibits greater specificity for HER2 and antibody-dependent cell-mediated cytotoxicity. RC48, with its cleavable linker, can readily diffuse to neighboring tumor cells. Moreover, MMAE is released more effectively in tumor tissues, resulting in targeted cytotoxicity. This suggests that MMAE accumulates in tumor tissues and demonstrates enhanced tumor-specific cytotoxicity through RC48 degradation (Yu et al., 2022). In xenograft tumor models, RC48 exhibits superior antitumor activity compared to T-DM1 (Patelli et al., 2022). RC48 holds promise as a novel therapeutic approach for solid tumors that are HER2-positive, as it demonstrates manageable toxicity and significant potency. Additionally, it enhances clinical outcomes for patients with breast cancer with HER2 expression (Yao et al., 2015).

RC48 is currently the only HER2-targeted ADC with publicly available and comprehensive data in the context of UC. In a phase I study, the efficacy of RC48 was evaluated in patients with advanced solid cancer that overexpressed HER2. Within a cohort of 24 patients eligible for evaluation, anticancer activity was observed, resulting in an ORR of 33.3%. Notably, one patient with UC exhibited a partial response (Gong et al., 2018). Moving on to a phase II study involving RC48, HER2-positive patients with locally advanced or mUC were enrolled (Sheng et al., 2019). These patients received RC48 treatment as a standalone intervention, administered via intravenous infusion at a dose of 2 mg/kg every 2 weeks, until disease progression, intolerable toxicity, voluntary withdrawal, or study termination. Out of a total of 43 patients, RC48 demonstrated a clinically significant ORR of 60.5% and a disease control rate (DCR) of 90.7% (39/43) in pretreated HER2-positive mUC patients, which includes individuals who had previously shown inadequate response to immunotherapy.

A study was conducted to assess the efficacy and safety of RC48 in patients with locally advanced or mUC who were negative for the HER2 receptor. A total of nineteen patients were enrolled in the study, with six patients classified as HER2-negative (Immunohistochemistry, IHC 0) and thirteen patients classified as HER2-negative (IHC 1+). The study reported an ORR of 26.3% and a DCR of 94.7% (18 out of 19 patients). These findings demonstrate the safety and effectiveness of RC48-ADC in patients with locally advanced or metastatic UC who do not express the HER2 receptor (Xu et al., 2022).

There are currently five phase II clinical trials underway, actively recruiting participants. The study NCT04879329 aims to determine the effectiveness of RC48, either alone or in combination with pembrolizumab, in treating HER2-positive UC. The phase II study NCT05495724 focuses on examining NMIBC, specifically evaluating the safety and efficacy of combining RC48 with tislelizumab as a treatment for patients with high-risk NMIBC that overexpresses HER2 (IHC 2+ or 3+). Tislelizumab is a humanized IgG4 anti-PD-1 monoclonal antibody designed to minimize binding to FcγR on macrophages. In this study, RC48 was administered intravenously at a dose of 120 mg on day 1, in combination with tislelizumab given at a dose of 200 mg on day 2, repeated every 3 weeks for three or four cycles, followed by transurethral resection biopsy. The ongoing study NCT05837806 also evaluates the efficacy and safety of combining tislelizumab and RC48. This study enrolled 27 participants with high-risk upper tract urothelial carcinoma that is HER2-positive. Another study, NCT03809013, focuses on investigating the efficacy and safety of intravenous RC48 in patients with locally advanced or metastatic UC that overexpresses HER2. Sixty participants will be treated with RC48 at a dose of 2.0 mg/kg once every 2 weeks until there is a loss of clinical benefit as assessed by the investigator, unacceptable toxicity, the decision of the investigator or participant to withdraw from therapy, or death. Finally, the study NCT04073602, which recruited 19 participants, is evaluating the efficacy and safety of RC48 for injection in subjects with metastatic or unresectable UC that does not overexpress HER2.

NCT05488353 is the sole phase IV trial investigating the combination of RC48 and penpulimab for the treatment of cisplatin-intolerant CT2-T4aNxM0 bladder UC patients. Once all screening activities are completed, eligible patients will receive neoadjuvant therapy. This therapy includes a 2.0 mg/kg RC48 injection on day 1, followed by 200 mg penpulimab injections every 21 days. The latter is administered as an intravenous infusion on day 1 of each cycle. Patients who undergo radical cystectomy after neoadjuvant therapy will be assessed for postoperative pathological complete remission rate, downstaging rate, and will receive regular follow-up for 2 years to evaluate the safety of the drug.

## Adverse events and toxicity of current clinical trials

The research on HER2-ADCs for UC is relatively limited, with most studies still in the phase II clinical trial. However, adverse events causing from these drugs have consistently been a focal point of concern, as they impact the clinical availability of ADCs. The following summarizes existing studies on adverse events of the treatment for UC using ADCs targeting HER2, serving as reference for evaluation.

In Xinan Sheng’s study (Sheng et al., 2019), 43 patients with HER2-possitive locally advanced or metastatic UC were treated with RC48. The median treatment duration for RC48 was 22 weeks, and all 43 patients experienced at least one treatment related adverse events (TRAEs). The most reported TRAEs were hypoesthesia (26, 60.5%), alopecia (24, 55.8%), leukopenia (24, 55.8%), fatigue (19, 44.2%), and neutropenia (18, 41.9%). About 25 patients (58.1%) experienced grade 3 TRAEs. The most reported grade 3 TRAEs were hypoesthesia (10, 23.3%) and neutropenia (6, 14.0%). No grade 4 or 5 TRAEs were observed. Eleven patients (25.6%) discontinue treatment due to severe TRAEs. In this study RC48 was well tolerated, since TRAEs of RC48 are commonly observed and well managed in clinical pratice.

In NCT03523572, primary results were analyzed and reported (Galsky et al., 2022). Thirty-four patients with HER2-possitive advanced/metastatic UC received combination of T-DXd and nivolumab. In the treatment duration (median 3.2 months for T-DXd and 4.1 months for nivolumab), TRAEs were seen in all patients. Grade ≥3 events were observed in 73.5% of all patients (44.1% related to T-DXd). TRAEs leading to drug discontinuation occurred in 32.4% of all patients (17.6% related to T-DXd). The most reported TRAEs were nausea (73.5%), fatigue (52.9%), and vomiting (44.1%). Although the combination of T-DXd and nivolumab shown anti-tumor activity, the safety of this treatment could not be ignored with the TRAEs associated with death is up to 20.4%.

Elisabeth de Vries’ study is based on NCT02999672 (de Vries et al., 2023), which enrolled 13 participants with advanced/metastatic UBC in cohort 1 and treated with single‐agent T‐DM1. Overall, 11 patients (84.6%) experienced grade≥1 any AEs. The most common TRAEs were pyrexia (5, 38.5%) and asthenia (4, 30.8%). Moreover, 7 patients (53.8%) experienced grade≥3 TRAEs. Three patients (23.1%) experienced fatal AEs. No AEs associated with death was considered to be related to T‐DM1, in another aspect, the reported AEs in this study were compatible with the known safety of T‐DM1.

## Conclusion

For patients with locally advanced and metastatic UC, current first line treatment options include platinum-based chemotherapy, checkpoint inhibitor immunotherapy and targeted therapies. Cisplatin-based chemotherapies are the prior candidates, and compare to single-agent cisplatin, cisplatin-based combination chemotherapy is associated with better objective response rate (ORR) and overall survival (OS).

Considering the toxicity associated with cisplatin, alternative combination therapy regimens are recommended for patients with a good performance status (World WHO/ECOG performance status <2) who are not suitable for cisplatin-based treatment. Gemcitabine plus carboplatin or pembrolizumab plus enfortumab vedotin are both popular options, but their overall benefits has not been fully assessed. The optimal regimen has not been well established, and further clinical randomized studies comparing these agents or development of neoadjuvant drugs are needed.

ADCs have emerged as a promising therapeutic option with demonstrated efficacy in various types of cancer, including UC. In recent years, significant advancements have been made in targeted therapies for UC, owing to a deeper understanding of the molecular and genomic characteristics of the disease. These breakthroughs have translated into encouraging outcomes in clinical trials, particularly in terms of OS, revolutionizing the approach to treating UC patients. The advent and regulatory approval of ADCs, such as enfortumab vedotin and sacituzumab govitecan, have brought about remarkable innovations in second-line therapy for UC. Moreover, HER2 has emerged as an effective target in multiple solid tumors and carcinomas, presenting a promising avenue for UC treatment. Ongoing phase II and phase III clinical trials have generated impressive results, further validating the potential of ADCs in targeting HER2. In the study of NCT03523572, combination treatment of T-DXd and nivolumab demonstrate that ORR was 36.7%, median progression-free survival (PFS) was 6.9 months, and median OS was 11 months. Nivolumab is being investigated as first-line therapy in combination with other drugs such as ipilimumab, and its utility as second-line therapy has been confirmed by multiple studies (Sharma et al., 2017; Sharma et al., 2019). In this case, it could be expected that the combination of T-DXd and nivolumab serves as new candidates for UC treatment. Current trials are focusing on assessing the efficacy and safety of single-agent usage of HER2 ADCs. However, the prevailing clinical guidelines for first-line treatment in UC emphasize combination therapy, which has demonstrated superior ORR and OS. Consequently, investigating the combination of HER2 ADCs with conventional chemotherapy agents for the treatment of advanced/malignant UC is poised to be a pivotal focus in the future. This avenue of research holds great promise as it aims to address the limitations of single-agent therapy and explore the potential synergistic effects of HER2 ADCs when used in combination with established chemotherapeutic agents. By combining these therapies, researchers aspire to enhance treatment outcomes and provide more effective options for patients with UC. While there are still numerous challenges to overcome, ADCs that target HER2 continue to hold promise as adjuvant therapeutic options across all stages of UC.

## Abbreviations

ADCC, Antibody-Dependent Cell-Mediated Cytotoxicity; ADCs, Antibody-Drug Conjugates; BOR, Best Overall Response; CR, Complete Response; DCR, Disease Control Rate; FDA, US Food and Drug Administration; HER2, Human Epidermal Growth Factor Receptor 2; mAbs, Monoclonal Antibodies; MMAE, Monomethyl Auristatin E; MUC, Metastatic Urothelial Carcinoma; NMIBC, Non-Muscle Invasive Bladder Carcinoma; ORR, Objective Response Rate; OS, Overall Survival; PFS, Progression-Free Survival; PR, Partial Response; RC48, Disitamab Vedotin; T-DM1, Trastuzumab Emtansine; T-Dxd, Trastuzumab Deruxtecan; TKIs, Tyrosine Kinase Inhibitors; TRAEs, Treatment Related Adverse Events; UC, Urothelial Carcinoma.
